# miRSeq: A User-Friendly Standalone Toolkit for Sequencing Quality Evaluation and miRNA Profiling

**DOI:** 10.1155/2014/462135

**Published:** 2014-06-24

**Authors:** Cheng-Tsung Pan, Kuo-Wang Tsai, Tzu-Min Hung, Wei-Chen Lin, Chao-Yu Pan, Hong-Ren Yu, Sung-Chou Li

**Affiliations:** ^1^Institute of Bioinformatics and Systems Biology, National Chiao Tung University, Hsinchu, Taiwan; ^2^Department of Medical Education and Research, Kaohsiung Veterans General Hospital, Kaohsiung, Taiwan; ^3^Genomics & Proteomics Core Laboratory, Department of Medical Research, Kaohsiung Chang Gung Memorial Hospital and Chang Gung University College of Medicine, Kaohsiung, Taiwan; ^4^Department of Parasitology, National Cheng Kung University College of Medicine, Tainan, Taiwan; ^5^Departments of Pediatrics, Chang Gung Memorial Hospital-Kaohsiung Medical Center, Graduate Institute of Clinical Medical Science, Chang Gung University College of Medicine, Kaohsiung, Taiwan; ^6^Children's Hospital, 12th Floor, No. 123, Dapi Road, Niaosong District, Kaohsiung 83301, Taiwan

## Abstract

MicroRNAs (miRNAs) present diverse regulatory functions in a wide range of biological activities. Studies on miRNA functions generally depend on determining miRNA expression profiles between libraries by using a next-generation sequencing (NGS) platform. Currently, several online web services are developed to provide small RNA NGS data analysis. However, the submission of large amounts of NGS data, conversion of data format, and limited availability of species bring problems. In this study, we developed miRSeq to provide alternatives. To test the performance, we had small RNA NGS data from four species, including human, rat, fly, and nematode, analyzed with miRSeq. The alignments results indicate that miRSeq can precisely evaluate the sequencing quality of samples regarding percentage of self-ligation read, read length distribution, and read category. miRSeq is a user-friendly standalone toolkit featuring a graphical user interface (GUI). After a simple installation, users can easily operate miRSeq on a PC or laptop by using a mouse. Within minutes, miRSeq yields useful miRNA data, including miRNA expression profiles, 3′ end modification patterns, and isomiR forms. Moreover, miRSeq supports the analysis of up to 105 animal species, providing higher flexibility.

## 1. Introduction

MicroRNAs (miRNAs) are non-protein coding RNAs. The mature products of miRNA genes are approximately 22-nt RNA fragments, rather than polypeptides. Because of intramolecular base pairing, the full-length primary transcripts form a hairpin structure plus unpaired ends, which is processed by the Drosha complex, trimming out the unpaired ends and releasing the hairpin structure into cytoplasm. The hairpin structure is further processed by Dicer, trimming out the terminal loop and releasing the RNA duplex. Either one or both strands of the RNA duplex are incorporated into RISC, functioning as mature miRNAs. By complimentary base pairing with the 3′ UTR, miRNAs guide RISC to the target gene's mRNAs, downregulating the target gene through either mRNA degradation or translational repression [1].

In addition to the biogenesis mechanisms and novel miRNA identification, many studies have focused on the regulatory functions of miRNAs. Since miRNA was discovered and characterized in* C. elegans*, numerous studies have reported that miRNAs play regulatory functions in a wide range of biological activities. Typically, the miRNA regulatory roles in cancer pathogenesis are investigated. According to the previous studies, miRNAs may function as tumor repressors, repressing tumor growth, tumor cell migration, or tumor cell proliferation [2, 3]. MiRNA may also play the roles of onco-miR, promoting tumor growth or tumor cell migration [4, 5]. MiRNAs are also involved in development regulation, including axon regeneration [6], sarcomere formation [7], and embryo development [8]. Moreover, miRNAs also serve as biomarkers of numerous diseases, such as Alzheimer's disease [9], liver pathology [10], heart failure [11], and graft-versus-host disease [12].

With more and more miRNAs identified in model organisms, miRNA-related studies focus on investigating miRNA regulation in diseases. Such studies depend on determining miRNA expression profiles between libraries, treatment versus control or normal versus disease. Therefore, next-generation sequencing (NGS) is usually applied to sequence miRNA, providing not only qualitative but also quantitative measurement of miRNA expression. However, the sequence data produced using NGS platforms typically require large disk space and are, therefore, difficult to be analyzed by the traditional biological researchers.

Currently, there are several online web services available for small RNA NGS data analysis [13, 14]. The user must first submit the raw sequence data in a fastq file to the online services and then request an analysis job. However, submitting large amounts of sequence data dramatically increases the Internet workload. Without a broadband network, transferring sequence data is time-consuming and becomes impeded. Although collapsed sequence data in the fasta format is also acceptable, converting fastq format into fasta format is generally a difficult task for biological researchers. Moreover, the requested jobs are typically held in a queue on the server rather than analyzed immediately. The server analyzes the requested jobs according to a first-come-first-served rule, making it difficult to estimate when the jobs are completed. Additionally, the online web services generally allow users to analyze small RNA NGS data of only a few species. So, an alternative is required.

Therefore, we developed miRSeq as an alternative. miRSeq is compatible with Windows operating systems. Following step-by-step instructions, users can easily install and operate miRSeq. From the initial raw NGS data in fastq format, miRSeq can evaluate the sequencing quality regarding percentage of self-ligation read, read length distribution, and read category. Within minutes, miRSeq yields useful miRNA data, including miRNA expression profiles, 3′ end modification patterns, and isomiR forms. Moreover, miRSeq supports the analysis on up to 105 animal species, providing higher flexibility.

## 2. Materials and Methods

### 2.1. Data Resources

miRSeq classifies sequence reads into categories and determines miRNA expression profiles by mapping the sequence reads back to known annotated transcripts, downloaded as follows. The miRNA data belong to miRBase 20. The sequences of mRNAs and ncRNAs were derived from the RefSeq 60 [15]. The sequences of tRNAs were downloaded from the Genomic tRNA database [16]. The sequences of rRNAs were provided by the SILVA database [17]. The sequence reads not belonging to any of the previous classes were classified into the unknown class.

miRSeq was developed with Perl and is compatible with Windows operating systems. The user can download miRSeq package via https://docs.google.com/forms/d/1WMsHS8jlxL-k3cL_UHTEKQGssno4ru6CuJMsGbPOqtk/viewform. Then, the user should first install the Perl complier and related modules by following the instructions in the readMe file. miRSeq is composed of two modules, including readPro and readMap, both of which can operate independently.

### 2.2. readPro

**Figure 1:**
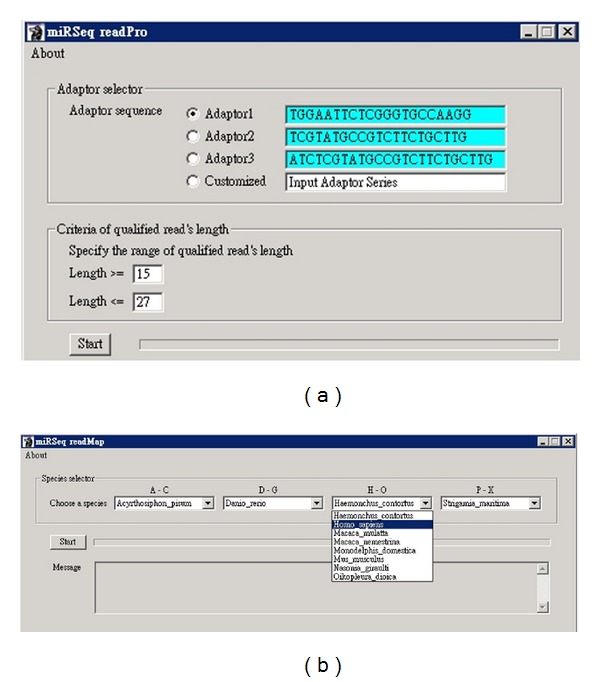
The operation interface of miRSeq. miRSeq is composed of readPro and readMap. (a) readPro deals with raw sequence reads in fastq format by collapsing raw reads into unique reads, tabulating read count, and trimming 3′ adaptor. (b) readMap is responsible for mapping the reads back to known annotations, classifying reads into different categories, determining miRNA expression profile, reporting 3′ end modification patterns, and analyzing isomiR forms.

The operation interface of readPro is illustrated in Figure 1(a). readPro deals with raw sequence reads in fastq format. readPro first collapses the raw reads into unique sequence tags with the read count of each unique sequence tag tabulated. Then, readPro trims the 3′ adaptor by referring to the sequence of the specified 3′ adaptor (Figure 1(a)). The sequence tags with 3′ adaptor detected and trimmed are named “clean reads” and further analyzed. However, the sequence tags without 3′ adaptor detected are discarded. The clean reads are further collapsed into unique clean reads and the read counts of those are also retabulated. readPro also analyzes the length distribution of the clean reads, by which the user can evaluate the sequence quality of the NGS data. Next, readPro sifts the qualified clean reads according to the user-specified length criteria (Figure 1(a)), and the qualified clean reads are presented in fasta format. In addition, only the unique clean reads with read count ≧2 are included in the fasta file for further analysis. The results of each readPro alignment are available.

### 2.3. readMap

The output result of readPro is the input data for readMap. The operation interface of readMap is illustrated in Figure 1(b). readMap maps the clean reads to pre-miRNAs with bowtie [18]. miRSeq collects the index data of miRNA and other annotated transcripts for 105 species (Supplementary Table 1 in Supplementary Material available online at http://dx.doi.org/10.1155/2014/462135). The miRNA expression profile, namely, the read count of each mature miRNA, can thereby be determined. The read count of miRNA is presented in transcript per million (TPM). In addition, the non-miRNA reads are further mapped in order to the rRNAs, tRNAs, mRNAs, ncRNAs, and genome (if applicable) of the specified species (Figure 1(b)). Thereby, clean reads are classified into categories. Using this information, users can evaluate whether the sample preparation protocol performed well.

When mapping reads back to pre-miRAs, variations preferentially occur at the 3′ terminal ends of the reads. Such variation could be caused by either RNA editing or nucleotide addition. It currently remains debatable which of the two accounts for the 3′ terminal variations. To bypass the debate, we named such variations “3′ end modifications.” Using the output of readMap, the users can compare the 3′ end modification patterns between libraries. MiRNA NGS reads usually exist as isomiRs, namely, miRNA isoforms exhibiting a length difference or position shift compared with the reference mature miRNAs. Previous studies have showed that isomiRs may perform regulatory functions between different libraries [19, 20]. readMap reports all isomiR forms of mature miRNAs. The results of each readPro alignment are also available.

### 2.4. Small RNA Sample Preparation

We prepared small RNA samples with the standard or nonstandard protocols. The standard small RNA sample preparation protocol applies in-gel size fraction to enrich the RNA fragments with size of approximately 22 nucleotides, excluding most of the non-miRNA fragments. For the L1 library (Table 1), we applied the nonstandard sample preparation protocol. During the in-gel size fraction step, we avoided extracting the RNA fragments exhibiting a length of approximately 22 nucleotides. Instead, we extracted the RNA fragments with a length adjacent to 22 nucleotides, picking up the RNA fragments with more diverse range in length. By doing so, we expect more non-miRNA contaminants are included.

## 3. Results

### 3.1. Library Summary

To illustrate the output data by miRSeq and to test the performance of miRSeq, we had small RNA NGS data of eight libraries from four species analyzed with miRSeq. The detailed information of the eight libraries is listed in Table 1. Among the eight libraries, L1, L2, L3, and L4 ones (from human and rat) are prepared and sequenced by our laboratory. To evaluate whether miRSeq can examine sequencing quality and detect sequencing quality variations between libraries, the L1 library was prepared with the nonstandard sample preparation protocol (Section 2) to apply variations between libraries. The remaining L2, L3, and L4 libraries were prepared with the standard sample preparation protocol. Then, the RNA samples were sequenced using the Illumina HiSeq platform. The L5, L6, L7, and L8 libraries (from nematode and fly) were downloaded from NCBI SRA database to examine miRSeq performance with public domain data [21]. When operating readPro (a tool of miRSeq package), the length constraint parameter was specified as 15 to 30.

### 3.2. Self-Ligation Reads

Small RNA samples are generally subject to 5′ adaptor and 3′ adaptor ligation at both ends before PCR amplification. Generally, the 5′ adaptor can ligate the 3′ adaptor without an RNA fragment inserted. Such self-ligation reads provide no useful information but garbage only, wasting sequencing consumables. As shown in Table 2, the self-ligation reads accounted for less than 1% in most libraries, except for L5 library (from* C. elegans*). Higher percentage of self-ligation read resulted in lower percentages of clean and qualified reads, leading to wasting budget. Thus, the percentage of self-ligation read is a crucial index for sequencing quality evaluation.

### 3.3. Length Distribution

**Figure 2:**
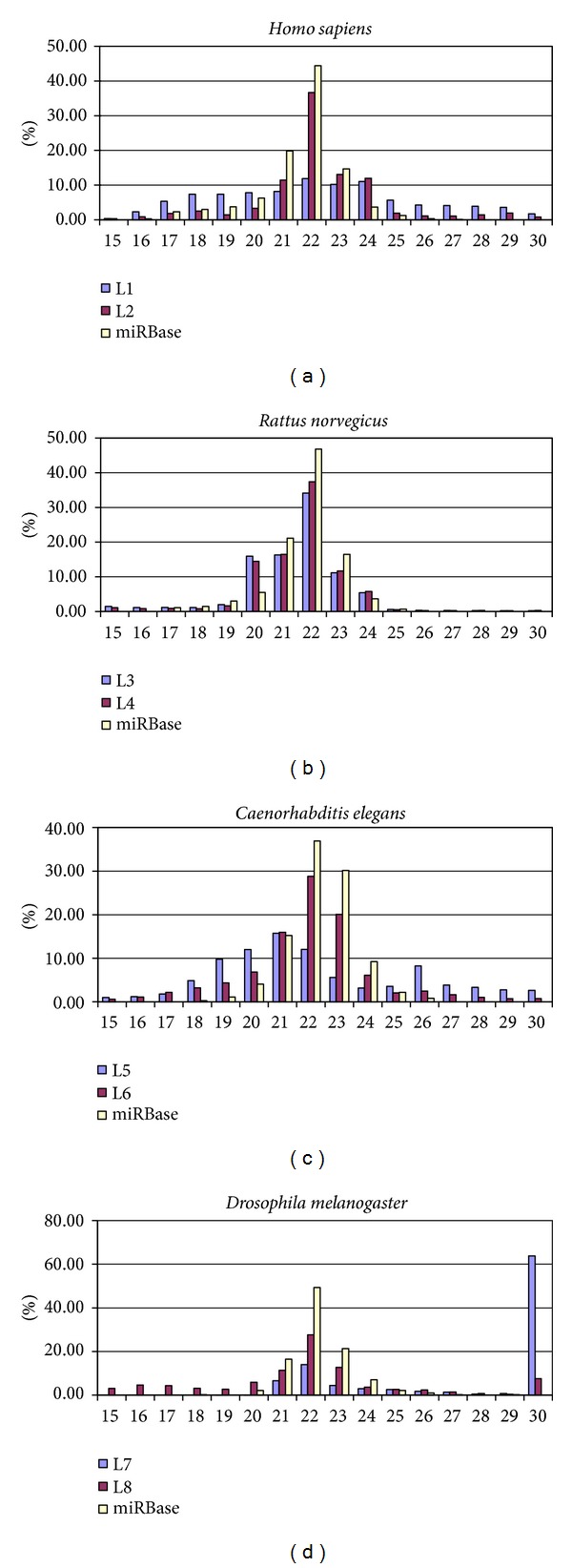
Length distribution comparisons of clean reads between libraries. From the output results of readPro, we may compare the length distribution of clean reads, examining if length enrichment occurs. (a) The length distribution pattern of the well-prepared L2 library was more similar to the one of miRBase miRNAs. (b) L3 and L4 libraries had similar distribution patterns. (c) The read length of L5 library scattered without enrichment. (d) The reads with length 30-nt dominated L7 library.

The lengths of animal miRNAs are highly enriched at nucleotides 21, 22, and 23. Therefore, the lengths of clean reads from a well-prepared library should also be highly enriched at nucleotides 21, 22, and 23 without even scattering. In Figure 2(a), the length distribution of L2 library's clean reads was highly enriched at 22-nt as miRBase miRNAs. However, the lengths of clean reads in L1 library scatter with much less enrichment, which is consistent with the experimental design of this study. The less enrichment of L1 library implied that L1 library contained more non-miRNA contaminants. Both L3 and L4 libraries were prepared according to the standard protocol, resulting in the same high enrichment as miRBase miRNAs (Figure 2(b)).

In Figure 2(c), different degree of length enrichments was also observed between L5 and L6 libraries. The result of L5 and L6 was much similar to the one of L1 and L2 in Figure 2(a). In Figure 2(d), although length enrichment was observed in L8 library, the enrichment was much less than other good examples such as L2, L3, L4, and L6. In addition, most clean reads in L7 library belong to the length 30-nt, implying more than 60% non-miRNA contaminants. The results of length distribution imply the proportions of miRNAs and can be confirmed in the next section.

### 3.4. Read Categories

**Figure 3:**
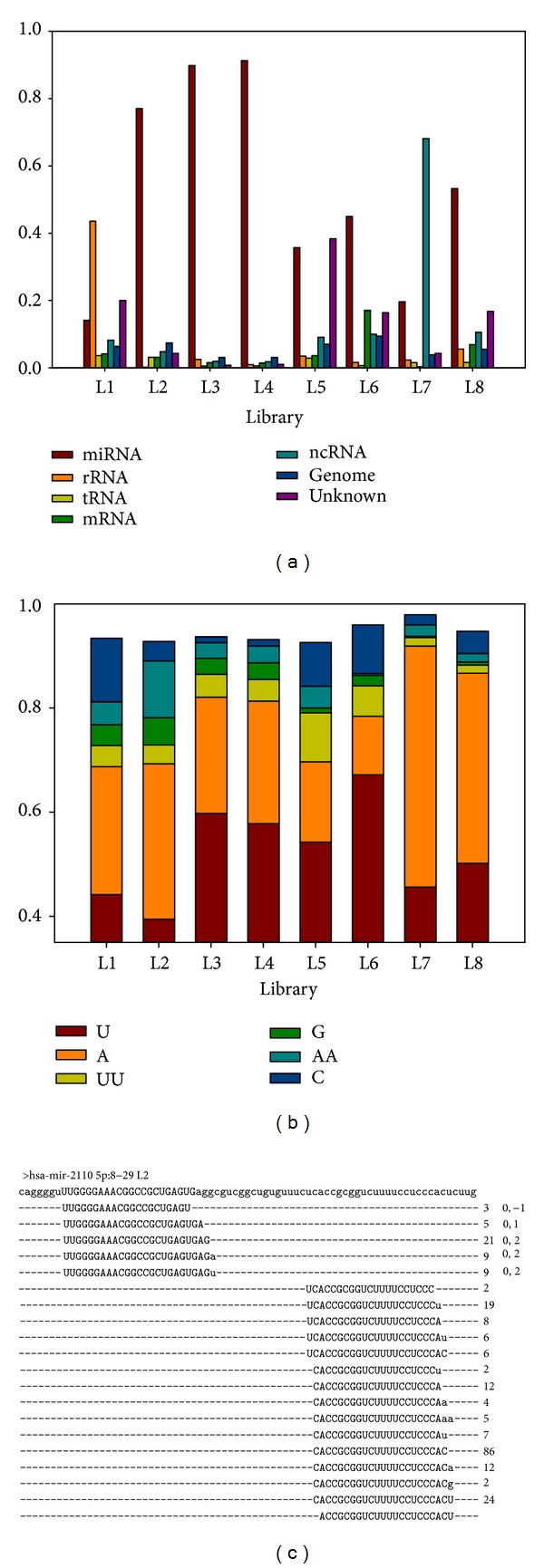
Illustration of readMap alignment output. From the output results of readMap, we may compare read category, 3′ end modification, and isomiR patterns among libraries. (a) readMap reports the read categories between libraries. High percentage of rRNA reads in L1 library resulted from the nonstandard protocol in the in-gel size fraction procedure. The non-miRNA reads accounted for much higher proportions in the libraries with less enriched read length at 22-nt. (b) readMap reports the 3′ modification patterns of miRNA reads. Such information is consistent between the libraries from the same species. Here, only the patterns more frequent than 1% are shown. (c) The top sequence denotes pre-miRNA sequence with mature miRNA marked in upper case. All isomiR forms are shown according to their relative position in pre-miRNA. The 3′ end modification patterns are presented in lower case. The middle digits and right-hand side information containing comma denote the read count and position shift of each isomiR.

At the small RNA sample preparation step, the in-gel size fraction is typically applied to remove contaminants from other RNA molecules. Thus, a high percentage of non-miRNA reads reflects the poor performance of the in-gel size fraction.  Figure 2 demonstrated high enrichment and low enrichment of clean reads' lengths. We further examined the categories of the clean reads, connecting length distribution and read category. As shown in Figure 3(a), miRNA dominated other molecules in L2 library. However, the miRNA reads accounted for only 16% in L1 library, much less than the rRNA reads did. Thus, the read category result was consistent with the experimental design of this study and also consistent with the result of the length distribution survey.

Consistent with length distribution, miRNA accounted for equal proportions in both L3 and L4 libraries. The lower miRNA proportion in L5 library resulted from higher proportion of unknown reads leading to less enrichment in length distribution. Finally, for L7 library, the reads with length 30 nucleotides belonged to ncRNAs, largely lowering down the proportion of miRNA reads. Comparing Figure 2 and Figure 3(a), the results of length distribution and read category were consistent with each other and both of them are critical indices for overall NGS quality. In summary, miRSeq is able to evaluate the sequencing quality of small RNA NGS data by using self-ligation read, length distribution, and read category.

### 3.5. miRNA Expression Profile

The major purpose of small RNA NGS is to acquire a miRNA expression profile. By mapping reads back to pre-miRNAs, readMap yields the miRNA expression profile. The expression profiles of the five most abundant miRNAs in libraries are illustrated in Table 3. The miRNA expression profile was presented in transcript per million (TPM) so that expression abundance could be compared between different libraries exhibiting unequal miRNA reads. The expression profiles of all miRNAs in libraries are available. In addition to expression profile, the number of detected pre-miRNA and mature miRNA was also provided, as shown in Table 4. Because L1 library exhibited considerably fewer miRNA reads than L2 library did (1.36 versus 7.63 millions), fewer pre-miRNAs and mature miRNAs were detected in L1 library. Such result resulted from and is consistent with the experimental design of this study. Thus, the results of the length distribution, read category, and miRNA numbers were consistent with each other.

### 3.6. 3′ End Modification Patterns

In addition to miRNA expression profiles, recently, 3′ end modification patterns of miRNA reads have also caught the attentions of researches [19]. miRSeq also provides the 3′ end modification patterns of miRNA reads. As shown in Figure 3(b), the 3′ end modification patterns between libraries from the same species were pretty similar. U dominated over other patterns in frequency, followed by A. In addition, AU-rich patterns accounted for more than 70% of all patterns.

### 3.7. IsomiRs Forms

When mapped back to pre-miRNA, miRNA reads are generally observed to exhibit position shift or length variation compared with reference mature miRNAs. Such a phenomenon is named “isomiR.” Previous studies using NGS data have showed that isomiRs performed specific regulatory functions [22, 23]. Therefore, all isomiR forms of mature miRNAs are provided by miRSeq. As illustrated in Figure 3(c), hsa-miR-2110 consisted of three isomiRs, the position shifts of which are shown. In addition, the 3′ end modification patterns are represented in lower case (i.e., a and u). According to miRBase 20, hsa-mir-2110 encodes mature miRNA only at the 5p arm, ranging from positions 8 to 29. However, applying NGS data, additional mature miRNAs can be detected at the opposite arm, named “opp-miRNA” in Table 4 [19, 23]. The opp-miRNA also consisted of isomiRs and 3′ end modification. Moreover, the newly identified opp-miRNAs could have higher expression abundance than the originally annotated miRNAs.

### 3.8. Time Needed for a miRSeq Alignment

miRSeq is compatible with Windows operating systems. To estimate how much time is needed for analyzing one library of NGS data, we processed the L1 NGS data by using miRSeq. As demonstrated in Table 5, miRSeq totally required 10 minutes to analyze 11 million NGS reads on the Windows 7 platform. Thus, the analysis of small RNA NGS data can be completed in little time. Even a large set of NGS data can be analyzed overnight.

## 4. Conclusion

To examine whether miRSeq can precisely evaluate sequencing quality, we prepared RNA samples well or poorly. The variation in in-gel size fraction resulted in several variations between the libraries, including the percentage of qualified reads, length distribution, and read category. The alignment results proved that miRSeq is competent for sequencing quality evaluation. To demonstrate the applicability of miRSeq in animal species, we analyzed the small RNA NGS data from a wide range of animal species, including primate, rodent, insect, and nematode. The alignment results proved that miRSeq is applicable for the 105 animal species. miRSeq is a user-friendly standalone toolkit for sequencing quality evaluation and miRNA profiling from NGS data. Following step-by-step instructions, users can easily install miRSeq and can then analyze small RNA NGS data on their own PC or laptop.

## 5. Discussion

With the prevalence of NGS application on miRNA study, more and more toolkits, including commercial and free ones, were developed for small RNA NGS data analysis. The current free toolkits are based on online web services. So, the user must first either submit the raw sequence data in fastq format or convert the raw data into the collapsed fasta format, which usually increases the workload of internet connection and biological researchers. With the progress of PC and laptop's equipment, analyzing small RNA NGS data in PC or laptop is now applicable. miRSeq provides an alternative for the analysis and quality evaluation of small RNA NGS data, saving the network connection and biological researchers much workload and troubles.

When operating readPro, the sequence of 3′ adaptor must be specified. There are several versions of 3′ adaptor available, confusing the users. In addition, the people preparing the small RNA samples could design their own special 3′ adaptor, making it difficult to choose the correct 3′ adaptor. The user may first use a small fraction of the fastq file for test, for example, the first 10,000 records. The 3′ adaptor by referring to which readPro yields higher proportion of clean reads is usually the correct choice. Otherwise, the user may also refer to the introduction information in the corresponding SRA page for the information of 3′ adaptor. In addition, the user may also consult the people generating the NGS data.

The time needed for each miRSeq alignment depends on the size of input data and PC or laptop's CPU and memory. Of course, the faster the CPU, the shorter the time. For memory, we strongly suggest that the memory installed be double the size of input data to avoid IO errors.

## Supplementary Material

We collected the information of known annotated transcripts, including pre-miRNAs, rRNA, tRNA, mRNA and ncRNA, of 105 animal species. The numbers of the transcripts are tabulated.

## Figures and Tables

**Table 1:** The detailed information of analyzed libraries. SRA in the “Source” column denotes that the small RNA libraries were downloaded from NCBI SRA database. The SRA IDs of the libraries were provided. CGMH denotes that the libraries were prepared and sequenced by our research team in Kaohsiung Chang Gung Memorial Hospital.

Library	Organism	Source	Details
L1	*Homo sapiens *	CGMH	Human breast cancer cell line: MDA-MB-361
L2	*Homo sapiens *	CGMH	Human prostate cancer cell line: PC3
L3	*Rattus norvegicus *	CGMH	Normal lung tissue from 4-month rat
L4	*Rattus norvegicus *	CGMH	Normal lung tissue from 4-month rat
L5	*Caenorhabditis elegans *	SRA: SRR1175721	Synchronized adult population of nematodes
L6	*Caenorhabditis elegans *	SRA: SRR1139598	Stage-matched population of nematodes
L7	*Drosophila melanogaster *	SRA: SRR513989	Abdomens and thoraxes from w1118 male flies infected by Nora virus
L8	*Drosophila melanogaster *	SRA: SRR351332	Whole bodies of 2-3-day-old wild-type flies

**Table 2:** Alignment results of readPro. The small RNA NGS data of eight libraries were analyzed with miRSeq. Raw reads were classified into clean, nonclean, or self-ligation after 3′ adaptor trimming step. The clean reads following specified criteria are classified as qualified reads for further analysis. The sequences of adaptors 1, 2, and 3 are TGGAATTCTCGGGTGCCAAGG, TCGTATGCCGTCTTCTGCTTG, and ATCTCGTATGCCGTCTTCTGCTTG, respectively. The sequence of adaptor C (CTGTAGGCACCATCAATCGT) is based on the information in the corresponding SRA page.

Library	All	Self-ligation	Nonclean	Clean	Qualified	Adaptor version
L1	11,229,160	0.72%	4.65%	94.62%	86.15%	Adaptor 1
L2	11,501,087	0.02%	3.34%	96.65%	86.12%	Adaptor 1
L3	6,314,030	0.04%	3.94%	96.02%	85.37%	Adaptor 1
L4	6,235,528	0.03%	3.71%	96.26%	87.98%	Adaptor 1
L5	22,634,033	15.08%	7.59%	77.33%	68.37%	Adaptor 2
L6	9,023,339	0.23%	3.73%	96.04%	74.08%	Adaptor 1
L7	10,314,488	0.00%	13.61%	86.39%	83.08%	Adaptor C
L8	22,435,248	1%	7%	92%	83%	Adaptor 3

**Table 3:** MiRNA expression profile. MiRNA expression profiles of libraries were presented in the unit transcript per million (TPM). Here, only the data of the five most abundant miRNAs is shown.

Library	1st miRNA	2nd miRNA	3rd miRNA	4th miRNA	5th miRNA	%
L1	hsa-miR-30a-5p	hsa-miR-21-5p	hsa-miR-181a-5p	hsa-miR-92a-3p	hsa-miR-22-3p	51.69%
L2	hsa-miR-92a-3p	hsa-miR-22-3p	hsa-miR-143-3p	hsa-miR-10a-5p	hsa-miR-21-5p	46.41%
L3	rno-miR-143-3p	rno-miR-30a-5p	rno-miR-26a-5p	rno-miR-181a-5p	rno-miR-22-3p	52.77%
L4	rno-miR-143-3p	rno-miR-30a-5p	rno-miR-26a-5p	rno-miR-22-3p	rno-miR-10a-5p	51.43%
L5	cel-miR-58-3p	cel-miR-70-3p	cel-miR-71-5p	cel-miR-65-5p	cel-miR-241-5p	83.53%
L6	cel-miR-80-3p	cel-miR-35-3p	cel-miR-52-5p	cel-miR-72-5p	cel-miR-229-5p	38.84%
L7	dme-miR-1-3p	dme-miR-317-3p	dme-miR-276a-3p	dme-miR-263a-5p	dme-miR-184-3p	63.56%
L8	dme-miR-1-3p	dme-miR-8-3p	dme-miR-184-3p	dme-let-7-5p	dme-miR-263a-5p	86.06%

**Table 4:** The numbers of detected miRNAs and pre-miRNAs. The values in brackets denote the numbers of mature miRNAs and pre-miRNAs of the corresponding species according to miRBase 20 annotation. In addition to the individual miRNA expression profile, the information of all miRNA profiles is also provided.

Category	Detected miRNA	Detected pre-miRNA	Detected opp-miRNA
L1	533 (2,578)	411 (1,872)	28
L2	1098 (2,578)	824 (1,872)	86
L3	425 (728)	272 (449)	15
L4	444 (728)	288 (449)	15
L5	229 (368)	167 (223)	10
L6	259 (368)	165 (223)	9
L7	257 (426)	158 (238)	4
L8	269 (426)	167 (238)	6

**Table 5:** The time needed for a miRSeq alignment. Input data: L1 small RNA NGS data, totally 11,229,160 reads and accounting for approximately 1.7 GB disk space.

OS	CPU	Memory	readPro	readMap
32 bit Win. XP	Intel Pentium 4, 3.0 GHz	1.0 GB	6 min.	40 min.
32 bit Win. XP	Intel Atom D525, 1.0 GHz	3.0 GB	12 min.	52 min.
64 bit Win. 7	Intel Core i5, 1.7 GHz	4.0 GB	5 min.	4 min.
64 bit Win. Server 2008	Intel Xeon E5-2620, 2.0 GHz	16.0 GB	5 min.	7 min.
