# Supplementary material for: miRSeq: A User-Friendly Standalone Toolkit for Sequencing Quality Evaluation and miRNA Profiling
Source: Biomed Res Int. 2014 Jun 24;2014:462135. doi: 10.1155/2014/462135 (PMC4119685; doi:10.1155/2014/462135)
Supplement: Supplementary file 1 — We collected the information of known annotated transcripts, including pre-miRNAs, rRNA, tRNA, mRNA and ncRNA, of 105 animal species. The numbers of the transcripts are tabulated. [file 462135.f1.pdf]

Supplementary Table 1. Statistics of data resources. The numbers of annotated transcripts from 105 species are tabulated.

Species	# pre-miRNA	# rRNA	# tRNA	# mRNA	# ncRNA
Acyrthosiphon pisum	117	34	0	17,674	930
Aedes aegypti	101	11	0	16,785	0
Amphimedon queenslandica	8	3	0	9,814	94
Anolis carolinensis	282	2	0	16,533	236
Anopheles gambiae	67	6	422	0	0
Apis mellifera	218	72	0	10,607	1,245
Artibeus jamaicensis	19	0	0	0	0
Ascaris suum	97	8	0	0	0
Ateles geoffroyi	60	0	0	0	0
Bombyx mori	489	10	0	15,121	620
Bos taurus	798	3	4,161	32,210	2,371
Branchiostoma belcheri	118	0	0	0	0
Branchiostoma floridae	156	9	0	28,623	0
Brugia malayi	134	9	0	11,460	0
Caenorhabditis brenneri	214	6	1,096	0	0
Caenorhabditis briggsae	177	4	958	19,404	0
Caenorhabditis elegans	223	7	820	26,066	23,837
Caenorhabditis remanei	156	5	971	31,476	0
Canis familiaris	324	0	906	0	0
Capitella teleta	124	4	0	0	0
Cerebratulus lacteus	2	3	0	0	0
Ciona intestinalis	348	5	0	14,181	625
Ciona savignyi	27	10	0	0	0
Cricetulus griseus	200	0	0	21,607	438
Culex quinquefasciatus	74	20	0	18,883	0
Cyprinus carpio	134	3	0	0	0
Danio rerio	346	17	12,292	27,226	1,054
Daphnia pulex	44	8	0	0	0
Drosophila ananassae	76	2	0	15,070	519
Drosophila erecta	81	8	0	15,048	568
Drosophila grimshawi	82	0	0	14,986	430

Drosophila melanogaster	238	18	304	27,749	1,616
Drosophila mojavensis	71	3	0	14,595	396
Drosophila persimilis	75	17	0	16,878	474
Drosophila pseudoobscura	210	3	0	0	0
Drosophila sechellia	78	38	0	16,471	579
Drosophila simulans	136	3	0	15,415	498
Drosophila virilis	74	10	0	14,491	654
Drosophila willistoni	77	30	0	15,513	496
Drosophila yakuba	80	11	0	16,082	521
Echinococcus granulosus	23	2	0	0	0
Echinococcus multilocularis	22	0	0	0	0
Equus caballus	341	3	503	20,675	700
Fugu rubripes	129	0	0	0	0
Gallus gallus	734	2	242	32,181	4,853
Glottidia pyramidata	1	3	0	0	0
Gorilla gorilla	322	5	0	70	0
Haemonchus contortus	187	1	0	0	0
Haliotis rufescens	5	0	0	0	0
Heliconius melpomene	92	6	0	0	0
Hippoglossus hippoglossus	40	0	0	0	0
Homo sapiens	1,872	29	631	37,246	11,679
Hydra magnipapillata	17	17	0	16,989	364
Ictalurus punctatus	281	6	0	1,288	0
Ixodes scapularis	49	6	0	20,467	0
Lagothrix lagotricha	48	0	0	0	0
Lemur catta	16	0	0	0	0
Leucosolenia complicata	1	0	0	0	0
Locusta migratoria	7	0	0	0	0
Lottia gigantea	60	1	0	0	0
Macaca mulatta	615	3	379	27,091	3,053
Macaca nemestrina	74	0	0	0	0
Macropus eugenii	3	3	0	0	0
Manduca sexta	94	0	0	0	0
Marsupenaeus japonicus	6	0	0	0	0

Monodelphis domestica	460	3	0	19,073	438
Mus musculus	1,186	6	433	29,707	6,060
Nasonia giraulti	32	2	0	0	0
Nasonia longicornis	28	0	0	0	0
Nasonia vitripennis	53	8	0	12,927	272
Nematostella vectensis	49	44	0	24,780	0
Oikopleura dioica	66	7	0	243	0
Ornithorhynchus anatinus	396	5	0	17,183	469
Oryzias latipes	168	3	0	22,084	391
Ovis aries	105	0	0	22,733	251
Pan paniscus	88	3	0	29,610	3,344
Pan troglodytes	656	8	463	35,265	4,114
Panagrellus redivivus	199	4	0	0	0
Paralichthys olivaceus	20	0	0	0	0
Petromyzon marinus	244	9	0	0	0
Pongo pygmaeus	634	0	471	0	0
Pristionchus pacificus	124	5	0	0	0
Pygathrix bieti	11	0	0	0	0
Rattus norvegicus	449	8	444	29,665	2,329
Rhipicephalus microplus	24	2	0	0	0
Saccoglossus kowalevskii	91	4	0	12,571	283
Saguinus labiatus	42	0	0	0	0
Sarcophilus harrisii	66	1	0	20,505	904
Schistosoma japonicum	55	29	0	0	0
Schistosoma mansoni	20	13	0	12,845	0
Schmidtea mediterranea	148	14	0	0	0
Strigamia maritima	3	3	0	0	0
Strongylocentrotus purpuratus	61	1	1,068	22,722	361
Sus scrofa	280	2	0	24,555	1,338
Sycon ciliatum	1	1	0	0	0
Symphalangus syndactylus	11	0	0	0	0
Taeniopygia guttata	246	1	0	15,568	491
Terebratulina retusa	1	3	0	0	0
Tetranychus urticae	52	15	0	0	0

Tetraodon nigroviridis	132	9	0	0	0
Tribolium castaneum	220	2	0	9,916	291
Xenopus laevis	22	0	0	11,034	18
Xenopus tropicalis	189	0	0	0	0
Xenoturbella bocki	8	1	0	0	0
